# Time-updated Fibroblast Growth Factor 23 Is Predictive for Posttransplant Diabetes Mellitus in Kidney Transplant Recipients

**DOI:** 10.1210/jendso/bvae055

**Published:** 2024-04-04

**Authors:** Amarens van der Vaart, Daan Kremer, Tessa Niekolaas, Stephan J L Bakker, Peter R van Dijk, Martin H de Borst

**Affiliations:** Department of Internal Medicine, Divisions of Nephrology, University Medical Center Groningen, University of Groningen, 9700 RB Groningen, the Netherlands; Endocrinology, University Medical Center Groningen, University of Groningen, Groningen, 9700 RB Groningen, the Netherlands; Department of Internal Medicine, Divisions of Nephrology, University Medical Center Groningen, University of Groningen, 9700 RB Groningen, the Netherlands; Department of Internal Medicine, Divisions of Nephrology, University Medical Center Groningen, University of Groningen, 9700 RB Groningen, the Netherlands; Department of Internal Medicine, Divisions of Nephrology, University Medical Center Groningen, University of Groningen, 9700 RB Groningen, the Netherlands; Endocrinology, University Medical Center Groningen, University of Groningen, Groningen, 9700 RB Groningen, the Netherlands; Department of Internal Medicine, Divisions of Nephrology, University Medical Center Groningen, University of Groningen, 9700 RB Groningen, the Netherlands

**Keywords:** fibroblast growth factor 23, post-transplant diabetes mellitus, kidney transplant recipients

## Abstract

**Objective:**

This work aimed to study whether fibroblast growth factor 23 (FGF23) is predictive for incident posttransplant diabetes mellitus (PTDM) in kidney transplant recipients (KTRs).

**Methods:**

We repeatedly analyzed plasma C-terminal FGF23 concentrations in 170 KTRs enrolled in the TransplantLines Biobank and Cohort Study. Associations of time-updated plasma FGF23 with incident PTDM were studied by Cox regression.

**Results:**

A total of 170 KTRs (46% female, aged 54.4 ± 12.4 years) with 540 FGF23 measurements were included. Plasma FGF23 concentrations at transplantation were 31.1 (0.76-2576) pmol/L. During a follow-up of 24 (12-24) months, 38 patients developed PTDM. The highest FGF23 tertile (compared to the lowest) was associated with an increased risk for PTDM (fully adjusted hazard ratio 20.9; 95% CI, 3.4-130.0; *P* < .001).

**Conclusion:**

In KTRs without diabetes at baseline, the highest tertile of FGF23, compared to the lowest, is predictive for development of PTDM.

Kidney transplant recipients (KTRs) are at risk of developing posttransplant diabetes mellitus (PTDM) with a prevalence of up to 30% to 50% [1, 2]. KTRs who develop PTDM have a higher risk of cardiovascular disease, infections, graft failure, and mortality [2]. Risk factors for PTDM are similar to those for type 2 diabetes, but additional transplant-related factors, such as use of immunosuppressive agents and past (viral) infections, contribute to the excess risk [3].

Fibroblast growth factor 23 (FGF23), a major phosphate-regulating hormone, is elevated in individuals with chronic kidney disease [4]. FGF23 levels tend to normalize in most patients after successful kidney transplantation, although levels remain elevated in some patients [5]. Prior studies have linked abnormal FGF23 levels with deregulated insulin metabolism, including elevated levels of insulin, leptin, and resistin [6, 7]. Whether FGF23 is associated with risk of PTDM has not yet been investigated. Therefore, we aimed to investigate the association between FGF23 and incident PTDM, and assessed whether the association was independent of traditional and transplant-related risk factors for PTDM.

## Material and Methods

The present study was conducted in a prospective cohort of KTRs who underwent transplants at the University Medical Center Groningen in the Netherlands. Further details about the TransplantLines cohort study can be found elsewhere [8]. A total of 170 individuals without diabetes at transplantation and available repeated FGF23 data were included. The study protocol was approved by the institutional review board (METC 2014/077) in accordance with the Declaration of Helsinki.

### Outcomes

Diabetes was defined as 1) use of oral glucose-lowering drugs, glucagon-like peptide 1 analogues or insulin; or 2) a random glucose greater than or equal to 11.1 mmol/L; or 3) glycated hemoglobin A_1c_ (HbA_1c_) above 6.5% (or 48 mmol/L). Prediabetes was defined as 1) a random plasma glucose greater than or equal to 7.8 mmol/L or 2) an HbA_1c_ above 6.0% (or 42 mmol/L) [9].

Our main analysis was conducted in all KTRs without diabetes at transplantation with the end point incident PTDM. On sensitivity analysis for the composite end point incident posttransplant prediabetes or PTDM, we included all individuals without prediabetes at baseline (n = 164). In a second sensitivity analysis with end point PTDM, baseline was set at 3 months after transplantation and only plasma FGF23 measurements after transplantation were included.

### Measurements

Plasma glucose, HbA_1c_, and FGF23 levels were determined at fixed time points (before transplantation, 3, 6, 12, and 24 months after transplantation). EDTA-anticoagulated plasma samples were stored at −80 °C until analysis. C-terminal FGF23 levels were detected in EDTA with enzyme-linked immunosorbent assay (BI-20702 FGF23 (C-terminal) Biomedica, AB_2935690). The minimal detectable concentration for the FGF23 assay was 0.08 pmol/L, with an intra-assay (within-run) coefficient of variation of 12% or less and an interassay coefficient of variation of 10% or less.

### Statistical Analyses

To study the association between plasma FGF23 and incident PTDM, we performed multivariable Cox regression analyses with step-wise adjustment for confounders, including age, sex, plasma glucose, and HbA_1c_ at transplantation, body mass index (BMI), systolic blood pressure, high-density lipoprotein (HDL) cholesterol, estimated glomerular filtration rate (eGFR), C-reactive protein (CRP), plasma phosphate, parathyroid hormone (PTH), and immunosuppressive agents, such as prednisolone and tacrolimus. All covariates (except age, sex, glucose, and HbA_1c_) were treated as time-dependent covariates in the models (fixed time points before transplantation, 3, 6, and 12 months after transplantation). Missing data (<10%) were addressed with multiple imputation technique based on predictive mean matching with regression models. Hazard ratios were outlined including a 95% CI. We examined the risk of PTDM with and without inclusion of plasma FGF23 by testing for differences in Harrell's C-statistics and −2 log likelihood calculated with and without the inclusion of FGF23 in the model. In all analyses, a *P* value less than .05 was considered statistically significant. R version 3.2.3 was used for all statistical analyses.

## Results

**Figure 1.**
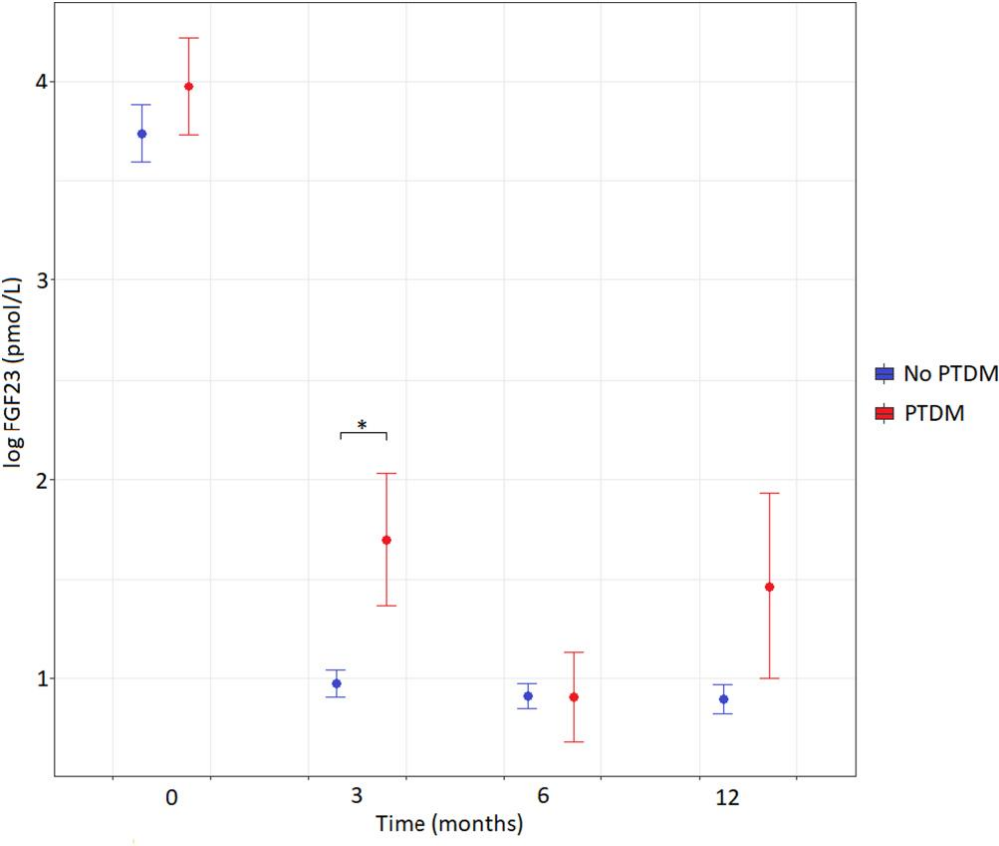
C-terminal plasma fibroblast growth factor 23 (FGF23) concentrations over time in individuals according to tertiles of baseline FGF23. Means with SEs are depicted.

A total of 170 KTRs (46% female, aged 54.4 ± 12.4 years) with 540 plasma FGF23 measurements were included in the present study. Plasma FGF23 concentrations at transplantation were 31.1 (0.76-2576) pmol/L, at 3 months 2.6 (0.4-68.4) pmol/L, at 6 months 2.3 (0.51-23.1) pmol/L, and at 12 months 2.3 (0.51-43.2) pmol/L. Participants who were in the highest tertile of FGF23 at baseline tended to also have higher levels at the subsequent measurements (at 3, 6, and 12 months) compared to those in baseline tertiles 1 or 2 (Fig. 1). Baseline HbA_1c_ levels were 5.4 ± 0.8% (35 ± 5 mmol/mol). Other baseline characteristics are presented in Table 1.

**Table 1.** Baseline characteristics

Characteristics	n = 170
Sex (female, %)	79 (46%)
Age, y	54.40 (12.35)
BMI	26.15 (3.91)
SBP, mm Hg	140 (20)
Glucose, mmol/L	5.4 (0.8)
HbA_1c_, mmol/mol	35 (5)
HDL cholesterol, mmol/L	1.20 (0.37)
PTH, mmol/L	26.60 (21.01)
Phosphate, mmol/L	1.36 (0.49)
eGFR, mL/min/1.73 m^2^	9.50 [6.79, 14.25]
Plasma FGF23, pmol/L	31.1 (0.76-2576)
Use of tacrolimus, %	39 (23)
Use of prednisolone, %	56 (33)
Donor type, n (%)	
Living donor	143 (84)
Donor	
Donation after brain death	15 (8)
Donation after circulatory death	12 (7)

Values are means (±SD), medians (interquartile range), or proportions (%). *P* values less than .05 were considered clinically significant and are presented in bold.

Abbreviations: BMI, body mass index; eGFR, estimated glomerular filtration rate; FGF23, fibroblast growth factor 23; HbA_1c_, glycated hemoglobin A_1c_, HDL, high-density lipoprotein; PTH, parathyroid hormone; SBP, systolic blood pressure.

**Figure 2.**
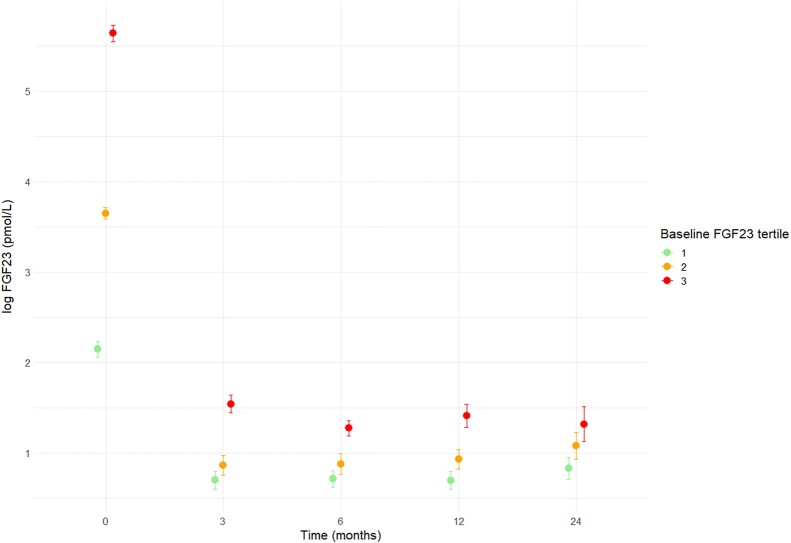
C-terminal plasma fibroblast growth factor 23 (FGF23) concentrations over time in individuals according to baseline FGF23 tertile. Means with SEs are depicted.

During a median follow-up of 24 months (range, 12-24 months) after transplantation, 38 (24%) patients developed PTDM. The course of plasma FGF23 levels over time is shown in Fig. 2. On Cox proportional-hazards regression analysis, being in the highest FGF23 tertile was associated with an increased risk of incident PTDM in the crude model and the fully adjusted model, as shown in Table 2. Harrell's C-index for the fully adjusted model without FGF23 and PTDM was 0.746, which was significantly improved to 0.766 after addition of FGF23 to the model (*P* < .01).

**Table 2.** Associations between time-updated fibroblast growth factor 23 and incident posttransplant diabetes mellitus

Events	Tertile 1 plasma FGF23: 0.40-2.23 pmol/L	Tertile 2 plasma FGF23: 2.24-7.73 pmol/L	Tertile 3 plasma FGF23: 7.73-2580 pmol/L	*P*
	2	8	28	
Crude	1.0 (ref)	4.0 (0.8-19.0)	**10.0 (2.0-51.0)**	**.01**
Model 1	1.0 (ref)	5.3 (1.1-26.3)	**18.5 (3.4-102.2)**	**<.001**
Model 2	1.0 (ref)	5.2 (1.1-25.9)	**19.8 (3.6-105.0)**	**<.001**
Model 3	1.0 (ref)	5.2 (1.0-26.2)	**17.5 (2.9-105.0)**	**.01**
Model 4	1.0 (ref)	5.0 (1.0-25.3)	**20.9 (3.4-130.0)**	**<.001**

Data are presented as hazard ratio plus 95% CI. *P* values less than .05 were considered clinically significant and are presented in bold.

Model 1: adjusted for age, sex, baseline HbA_1c_, and baseline glucose.

Time-updated covariates:

Model 2: model 1 plus use of prednisolone, use of tacrolimus, and use of vitamin D supplement.

Model 3: model 2 plus BMI and HDL cholesterol.

Model 4: model 3 plus eGFR, hs-CRP, plasma phosphate, and plasma PTH.

Abbreviations: BMI, body mass index; eGFR, estimated glomerular filtration rate; FGF23, fibroblast growth factor 23; HbA_1c_, glycated hemoglobin A_1c_, HDL, high-density lipoprotein; hs-CRP, high-sensitivity C-reactive protein; PTH, parathyroid hormone; ref, reference.

In a first sensitivity analysis, excluding individuals with prediabetes or diabetes at baseline, the highest tertile of FGF23 remained associated with the composite end point incident prediabetes or PTDM (Table 3). Given that plasma FGF23 concentrations strongly decrease after transplantation, as shown in Fig. 2, we performed a second sensitivity analysis including only plasma FGF23 measurements after transplantation. This analysis yielded similar results with borderline significance (Table 4).

**Table 3.** Associations between time-updated fibroblast growth factor 23 and composite end point incident prediabetes or posttransplant diabetes mellitus

Events	Tertile 1 plasma FGF23: 0.40-2.52 pmol/L	Tertile 2 plasma FGF23: 2.52-10.8 pmol/L	Tertile 3 plasma FGF23: 10.8-2580 pmol/L	*P*
	8	16	44	
Crude	Ref (1.0)	1.6 (0.7-4.0)	**2.7 (0.9-7.8)**	**.06**
Model 1	Ref (1.0)	1.8 (0.7-4.5)	**3.7 (1.2-11.4)**	**.02**
Model 2	Ref (1.0)	1.7 (0.7-4.3)	**3.5 (1.1-10.9)**	**.03**
Model 3	Ref (1.0)	1.6 (0.6-4.2)	**3.4 (1.1-10.7)**	**.03**
Model 4	Ref (1.0)	1.9 (0.7-5.4)	**4.1 (1.1-14.6)**	**.03**

Data are presented as hazard ratio plus 95% CI. *P* values less than .05 were considered clinically significant and are presented in bold.

Model 1: adjusted for age, sex, baseline HbA_1c_, and baseline glucose.

Time-updated covariates:

Model 2: model 1 plus use of prednisolone, use of tacrolimus, use of vitamin D supplement.

Model 3: model 2 plus BMI and HDL cholesterol.

Model 4: model 3 plus eGFR, CRP, plasma phosphate, and plasma PTH.

Abbreviations: BMI, body mass index; CRP, C-reactive protein; eGFR, estimated glomerular filtration rate; FGF23, fibroblast growth factor 23; HbA_1c_, glycated hemoglobin A_1c_, HDL, high-density lipoprotein; PTH, parathyroid hormone; Ref, reference.

**Table 4.** Associations between time-updated fibroblast growth factor 23 after transplantation and incident posttransplant diabetes mellitus

Events	Tertile 1 plasma FGF23: 0.40-1.86 pmol/L	Tertile 2 plasma FGF23: 1.86-3.22 pmol/L	Tertile 3 plasma FGF23: 3.22-68.4 pmol/L	*P*
	1	4	7	
Crude	Ref (1.0)	4.2 (0.5-37.3)	**7.1 (0.9-58.1)**	**.07**
Model 1	Ref (1.0)	4.3 (0.5-41.2)	**8.0 (0.9-69.8)**	**.06**
Model 2	Ref (1.0)	6.3 (0.7-61.2)	**10.6 (1.2-96.0)**	**.04**
Model 3	Ref (1.0)	8.2 (0.8-86.3)	**16.8 (1.6-178.0)**	**.02**
Model 4	Ref (1.0)	8.6 (0.7-100.0)	**18.0 (1.5-224.0)**	**.03**

Data are presented as hazard ratio plus 95% CI. *P* values less than .05 were considered clinically significant and are presented in bold.

Model 1: adjusted for age, sex, baseline HbA_1c_, and baseline glucose.

Time-updated covariates:

Model 2: model 1 plus use of prednisolone, use of tacrolimus, and use of vitamin D supplement.

Model 3: model 1 plus BMI and HDL cholesterol.

Model 4: model 1 plus eGFR, CRP, plasma phosphate, and plasma PTH.

Abbreviations: BMI, body mass index; CRP, C-reactive protein; eGFR, estimated glomerular filtration rate; FGF23, fibroblast growth factor 23; HbA_1c_, glycated hemoglobin A_1c_, HDL, high-density lipoprotein; PTH, parathyroid hormone; Ref, reference.

## Discussion

In this cohort of KTRs, individuals in the highest tertile of plasma FGF23 during the first 24 months post transplant had a higher risk of developing PTDM compared to those in the lowest tertile, independent of diabetes-related or transplant-related risk factors.

Although the observational design of this study precludes mechanistic explanations, we can speculate about potential mechanisms by which FGF23 may increase the risk of PTDM. FGF23^−/−^ ablated mice were found to be hypoglycemic, with increased peripheral insulin sensitivity and subcutaneous glucose tolerance [10]. Furthermore, mice with a PHEX mutation, leading to FGF23 overexpression, also displayed hyperglycemia and hypoinsulinemia, which are signs of (early) diabetes. While humans with a *PHEX* mutation, leading to X-linked hypophosphatemic rickets (XLH), have not been reported to be at higher risk of developing diabetes, this may have been understudied as XLH is very rare [11]. Furthermore, FGF23 is positively associated with markers of insulin resistance and adiposity [12-14]. Recently we found that a higher plasma FGF23 is associated with incident diabetes in the general population [15]. To our knowledge, the present study is the first to investigate the association between FGF23 and PTDM.

Strengths of the present study include the systematic and scheduled study visits at the time of transplantation (baseline), as well as 3, 6, 12, and 24 months post transplantation, enabling the use of time-updated plasma FGF23 measurements in all models. However, several limitations warrant mention. First, the relatively short follow-up period of 24 months after transplantation may have had the potential to underestimate hazards. Nevertheless, given the proportional high event rate observed, this is not likely to substantially affect our findings. Second, due to the observational nature of our study design, we cannot exclude the risk of residual confounding. Third, the reliability of HbA_1c_ measurements may be compromised in the initial 3 months following transplantation. Nonetheless, a sensitivity analysis employing a baseline set at 3 months post transplantation, which incorporated only plasma FGF23 measurements taken after transplantation, yielded comparable results. Fourth, oral glucose tolerance test–based criteria would be a better diagnosis for PTDM according to a consensus report [16], but unfortunately data were not available. Importantly, we were unable to measure alpha klotho levels in this study. We acknowledge this as a significant limitation and advocate for future studies to elaborate on the potential role of alpha klotho in the context of PTDM.

In summary, we found that a higher plasma FGF23 during the first 24 months after kidney transplantation was associated with a higher risk of PTDM. Our data provide a rationale for further studies on whether targeting FGF23 could improve insulin metabolism and reduce PTDM risk in KTRs.

## Data Availability

The participants of this study did not give written consent for their data to be shared publicly, so due to the sensitive nature of the research supporting data are not available.

## Abbreviations

BMI: body mass index
CRP: C-reactive protein
eGFR: estimated glomerular filtration rate
FGF23: fibroblast growth factor 23
HbA_1c_: glycated hemoglobin A_1c_
HDL: high-density lipoprotein
KTRs: kidney transplant recipients
PTDM: posttransplant diabetes mellitus
PTH: parathyroid hormone
